# Evaluating the Clinical Reasoning of Student Health Professionals in Placement and Simulation Settings: A Systematic Review

**DOI:** 10.3390/ijerph19020936

**Published:** 2022-01-14

**Authors:** Jennie Brentnall, Debbie Thackray, Belinda Judd

**Affiliations:** 1 Work Integrated Learning, Faculty of Medicine and Health, The University of Sydney, Sydney, NSW 2006, Australia; belinda.judd@sydney.edu.au; 2 Physiotherapy, School of Health Sciences, University of Southampton, Southampton SO17 1BJ, UK; d.thackray@soton.ac.uk

**Keywords:** clinical reasoning, medicine, nursing, allied health, students, assessment and evaluation

## Abstract

(1) Background: Clinical reasoning is essential to the effective practice of autonomous health professionals and is, therefore, an essential capability to develop as students. This review aimed to systematically identify the tools available to health professional educators to evaluate students’ attainment of clinical reasoning capabilities in clinical placement and simulation settings. (2) Methods: A systemic review of seven databases was undertaken. Peer-reviewed, English-language publications reporting studies that developed or tested relevant tools were included. Searches included multiple terms related to clinical reasoning and health disciplines. Data regarding each tool’s conceptual basis and evaluated constructs were systematically extracted and analysed. (3) Results: Most of the 61 included papers evaluated students in medical and nursing disciplines, and over half reported on the Script Concordance Test or Lasater Clinical Judgement Rubric. A number of conceptual frameworks were referenced, though many papers did not reference any framework. (4) Conclusions: Overall, key outcomes highlighted an emphasis on diagnostic reasoning, as opposed to management reasoning. Tools were predominantly aligned with individual health disciplines and with limited cross-referencing within the field. Future research into clinical reasoning evaluation tools should build on and refer to existing approaches and consider contributions across professional disciplinary divides.

## 1. Introduction

Systemic changes in healthcare are requiring graduates to be better prepared for work in diverse settings, in teams, and addressing increasingly chronic and complex healthcare needs [1]. To this end, graduates require competence in clinical reasoning; the process of ‘gathering and synthesising information; generating hypotheses; and formulating a clinical impression, diagnosis, prognosis, treatment, care, and/or management plan’ [2]. It is clinical reasoning that integrates the ‘cognitive, psychomotor and affective skills’ required to be ‘adaptive, iterative, and collaborative’ [3]. Therefore, the more autonomous and responsible the health professional, and the more dynamic and complex the situation (including technological advancements), the greater the need for clinical reasoning [4]. Accordingly, the development and evaluation of clinical reasoning is an increasing focus in health professional education [5,6,7]. It is also included as an essential graduate attribute in many health professional programs and a competency in many health professional frameworks internationally [7].

Despite the agreed importance of clinical reasoning, there is a lack of consensus on how it is conceptualised and the definitions of related terminology [2,3,4,6,7]. The term clinical reasoning (CR) is often used synonymously with terms such as decision making, critical thinking, problem solving, clinical judgement, and diagnostic reasoning [4,7]. It is also used as a ‘short hand’ for a broad concept [8], with variations in what comprises clinical reasoning by health professional discipline and also within disciplines [3]. The lack of consensus, along with the varied use of terminology, limits the advancement of education that will prepare graduates for multi-disciplinary teamwork in dynamic and complex situations inherent to healthcare.

There have been many attempts to define clinical reasoning across the professions and to find methods to teach and assess all the constructs related to the concept. Furthermore, there is a growing interest to know when a health professional student develops clinical reasoning, and there have been several recent systematic reviews of evaluations of the time frame of when students develop their clinical reasoning. Each of these reviews has taken a different focus. Specifically, Carter, Creedy, and Sidebotham [9] reviewed the tools used to measure ‘critical thinking’ development in undergraduate nursing and midwifery education. They identified tools by examining papers that applied an experimental design and measured critical thinking on multiple occasions in undergraduate nursing and midwifery education [9]. Similarly, Macauley et al. [10] systematically reviewed evaluations of clinical reasoning that were used as outcome measures, this time in examining simulation programs in any health profession. They also included broad outcome measures that did not necessarily focus on clinical reasoning (e.g., the physical therapy entry-level competency assessment, the Assessment of Physiotherapy Practice [10]). In a recent scoping review, Daniel et al. [5] expanded the scope to investigate approaches to clinical reasoning evaluation used in a range of settings (workplace-based, simulation-based, and non-workplace-based settings), rather than only outcomes-based research, but restricted their review to the evaluation of the clinical reasoning of medical students, residents, and physicians. Between these reviews [5,9,10] a wide range of measures have been identified, but only one review has extended beyond medicine and nursing [10]. The focus of these reviews has also generally been on research outcome measures [5,9]. Yet, there are a myriad of approaches to the evaluation of students that are suited to application in different educational contexts but not necessarily suited to use as research outcome measures [10].

Considering the need of education providers to identify their student health professionals’ proficient development of clinical reasoning in preparation for complex and uncertain work, there remains a need to establish means by which this may be evaluated. There exist dominant theories regarding the development of expertise in clinical reasoning—namely, Script Theory [11,12] describes the restructuring of knowledge as reasoning is practiced and reinforced with the development of expertise such that novices use knowledge networks to progress through detailed reasoning in a cognitively demanding process, whereas experts use ‘illness scripts’ to efficiently target information gathering and checking and arrive at a total solution. Alternately, clinical reasoning has been viewed as a skill to which Dreyfus and Dreyfus’ Model of Skill Development [13] applies, positing that novices are reliant on rules learned from others and skill progresses through a number of stages of the increasing capability to recognise patterns and handle uncertainty through to expertise where solutions are intuitively recognised. These models have informed the conceptualisation of clinical reasoning development in health professions education.

As clinical reasoning is a consequential capability (38), characterised by the integration of ‘cognitive, psychomotor and affective skills’ [3], and inherently requires flexible application, there is great interest in its development in experiential simulated and workplace-based settings, thereby evaluating it as an applied skill [14] or capability. Therefore, it is not a skill suited for evaluation in a classroom setting and does not focus on purely measuring a student’s cognitive capacity or knowledge base [15]. However, there has not been any research in this area of reasoning across disciplines. Further, to date, no research has synthesised the evidence on what the student evaluation tools on the topic of ‘clinical reasoning’ are purporting to evaluate, even in medicine and nursing. Including such details may provide a basis for furthering understanding of the constructs used and the relationships between them. Without this information, it is possible, perhaps likely, that a multitude of different aspects of clinical reasoning and associated constructs are being evaluated without cognisance of the boundaries and connections between them. With these clear gaps, the aim of this review is to systematically identify the tools used to evaluate clinical reasoning and determine the constructs the tools intend to assess.

The following research questions guided this review:

1. What tools have been developed or investigated for evaluating students’ clinical reasoning as applied in clinical education placement and simulation settings of health professional education?
2. What constructs or aspects of clinical reasoning are those tools designed to assess?

## 2. Materials and Methods

In a systematic approach, potentially eligible studies were identified by searching the databases of CINAHL (via EBSCO), ERIC, EMBASE, Medline and pre-Medline, PsychInfo (via Ovid), and Proquest Nursing and Allied Health. The search strategy (Supplemental Table S1) was inclusive of a wide range of allied health disciplines and medicine: audiology, dietetics, exercise physiology, medicine, nursing, occupational therapy, paramedicine, pharmacy, physiotherapy/physical therapy, podiatry, psychology, social work, speech/speech, and language pathology/therapy. Students were learners in their primary professional training (i.e., not post-professional continuing development or specialisation). The terms reasoning and the variants critical thinking, judgement, problem solving, and decision making were included, mapped to database subject headings as relevant. Likewise, the search included a range of terms pertaining to evaluation and measurement (e.g., assessment, inventory, test, scale, measure, index, survey, rubric, etc.), adjusted to database subject headings as relevant. Searches were limited to publication dates of 2000–2018, in English language only, and, where possible, to peer-reviewed sources. 

Citations were imported into Covidence (Melbourne, VIC, Australia) for management. Abstracts, and, where necessary, full-text papers, were each screened by one author according to the inclusion and exclusion criteria set out in Table 1. To ensure consistency within the team, at each stage papers were screened until several included studies were identified, and then, the team met to discuss the criteria for inclusion and exclusion until consensus and consistency were reached. Screening then continued, with even minor uncertainties discussed between the authors in regular meetings throughout the screening.

This review focussed on evaluations used in clinical placement or simulation settings. However, uncertainties arose during the screening process due to the incomplete or unclear reporting of relevant information within the papers. To resolve these uncertainties, a conservative approach was taken, whereby if the setting in which the student was evaluated was unclear in the paper, but the evaluation was reported as connected to student learning in clinical or simulation settings (i.e., eligible settings), the paper was included. Consensus for the other criteria was readily attained given the consistent reporting of these factors (e.g., focus on clinical reasoning or related concept, or outcomes) by authors.

Data extraction was completed by all authors and included authorship and year of publication, country or countries in which the study was undertaken, the tool or tools that were studied, aims, methods, the disciplines and levels of students and any other participants, the theoretical underpinning of the tool (as stated by the authors), and the construct evaluated (as stated by the authors). In cases in which papers included multiple evaluation tools, data were extracted for each relevant tool.

To ensure consistency, for each data extraction item, examples were discussed among the team and noted at the top of the data collection table prior to data extraction. Initially, data were extracted from two papers by different team members, and these were discussed among the team to reach consensus and clarity. Data from each eligible paper were then extracted by one team member per paper, working into a common data extraction table where the others’ work was visible for consistency. Uncertainties were discussed at regular meetings, with a second team member allocated to extract data from individual papers into a new table line in preparation for these meetings as required. Finally, all data for this report were reviewed against each paper by the first author.

## 3. Results

**Figure 1:**
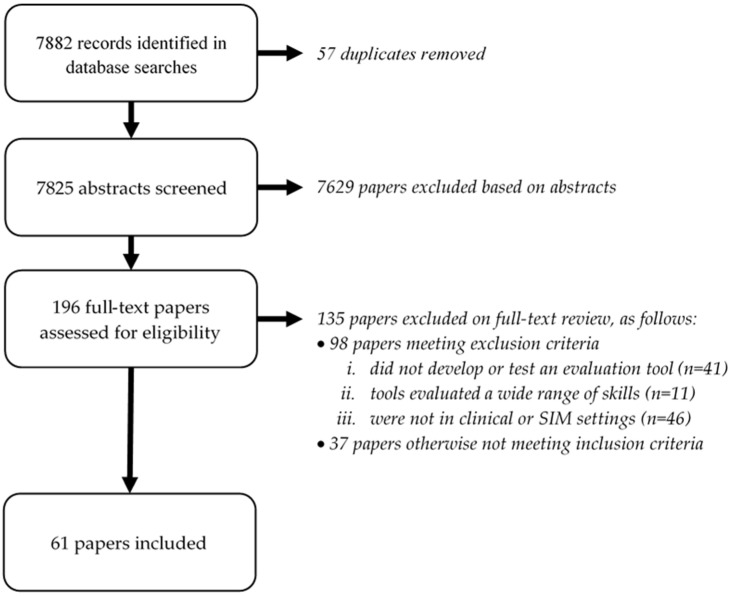
Flow diagram of search.

The 7882 records identified in database searches were narrowed to 61 included papers (Figure 1). Of the 196 papers that appeared to meet the criteria or were unclear from the abstract screening, 135 were excluded at full-text review, predominantly because the evaluation tools were used in settings such as university classes rather than in clinical- or simulation-based settings (n = 46), or because the paper did not report on the development or testing of an evaluation tool (n = 41).

### 3.1. Overview of Included Studies

The majority of the included 61 papers described studies within medicine and nursing, with 28 and 25 papers, respectively, plus one paper including participants from both of those professions. The remaining papers were in midwifery (n = 3), physical therapy (n = 2), occupational therapy (n = 1) and pharmacy (n = 1). Around half of the papers addressed the development and testing of the Script Concordance Test (SCT; n = 19; [16]) or the Lasater Clinical Judgement Rubric (LCJR; n = 13; [17]) and variants. The SCT is a test for predominantly diagnostic medical scenarios, where the examinee’s answers are scored based on the level of agreement with responses provided by a panel of experts [16]. The included studies each examine different case vignettes and expert decisions. The LCJR describes performance expectations, as well as language for feedback and assessment of predominantly nursing students’ clinical judgment development in a detailed and developmental rubric [17]. Several variants have been developed and tested. An overview of papers by evaluation tool is presented in Supplementary Table S2.

The papers were dominated by studies undertaken in the United States of America (n = 24), including 5 studies of the SCT and 7 studies of the LCJR. Other studies were undertaken in Canada (n = 8, including 5 on the SCT), Australia (n = 6), France (n = 5), Korea (n = 5, including 3 on LCJR variants), and a range of other countries. The distribution of publication years illustrates a trend of increasing publications on this topic over time, and particularly since 2015, considering that further papers published in 2018 would not have yet been in the databases at the time of the search.

### 3.2. Conceptual Foundations of the Clinical Reasoning Evaluation Tools

The included papers drew from a variety of conceptual frameworks (Table 2). Representing the topics identified in this review, the included papers were grouped into those stating they evaluate clinical decision making, clinical judgement, clinical reasoning, critical thinking, and situation awareness. The final category included papers that do not state a specific construct being measured, though they report measuring clinical reasoning and related constructs in general. Within each group, papers were arranged by the theoretical underpinning that was named in the paper. Evaluation tools for which there were no theoretical underpinnings identified appear at the end of each group.

Even within disciplines, there was a clear lack of agreement regarding critical thinking, with two different consensus statements in nursing [18,19], giving rise to several evaluation tools including the Carter Assessment of Critical Thinking in Midwifery [20,21,22,23,24,25] examining students’ critical thinking skills—a construct almost exclusively evaluated for nursing students. Another evaluation of critical thinking skills, through a clinical viva, was used for nursing students [26] and was derived by adapting a nursing competence assessment [27], while another competence assessment (the Physical Therapy Clinical Performance Instrument [28]) was indirectly adapted to create an evaluation of physical therapy interns’ clinical decision making [29]. The one evaluation of critical thinking skills used for medical students [30] was based on a problem-solving process [31] and piloted in high fidelity simulation.

Two evaluations utilised context-specific reasoning frameworks, both drawing on mnemonic devices to both guides and evaluate students. An occupational therapy clinical reasoning example [32] applied the A SECRET approach (Attention, Sensation, Emotion Regulation, Culture, Relationships, Environment, and Task [33]). In an example evaluating medical students’ clinical documentation [34], the IDEA framework (Interpretive summary, Differential diagnosis, Explanation of reasoning, and Alternative diagnosis with explanation) was combined with RIME descriptions (Reporter, Interpreter, Manager, Educator [35]).

Contrasting in specificity with the context-specific frameworks was the consideration of broad cognitive frameworks and abilities. Script Theory [11,12], which posits that expert clinical reasoning largely draws upon patterns, was used as the foundation of the Script Concordance Test [16] and variants including a multiple-choice examination [36] and written ‘think aloud’ test [37]. Situation awareness [38] was evaluated for nursing students [39]. Drawing upon an even more general foundation, a critical thinking skills evaluation [40] used for nursing students was based on Benner’s levels of nursing expertise, where students are considered to move through five levels of increasing proficiency, i.e., novice, advanced beginner, competent, proficient, and expert [41], and Bloom’s Taxonomy of Educational Objectives, where hierarchical models are used to classify educational learning objectives by levels of complexity and specificity [42]. Similarly, a clinical reasoning evaluation used for physical therapy students [43] was based on multiple sources of knowledge regarding clinical reasoning with evaluations based on the Revised Bloom’s Taxonomy of Educational Objectives [44] and the Dreyfus Model of Skill Acquisition [13] which describes skill development through instruction and experience of five developmental stages from novice to mastery. In another instance, authors reported the complementary use of a clinical reasoning model [45] and social cognitive theory [46,47], which considers that an individual’s thoughts and feelings, as well as the social environment, affect their own behaviour, to derive an evaluation of anxiety and confidence in clinical decision making [48].

Finally, across all the included papers, published models of clinical judgement [49], clinical decision making, and clinical reasoning processes [45,50,51] were cited as directly or indirectly underpinning the Lasater Clinical Judgement Rubric [17] and variants [52,53,54,55,56], as well as several evaluations of clinical decision making [48] and clinical reasoning [57,58]. These represented the most direct link between conceptual models and student evaluations.

Collectively, the identified frameworks represent a broad spectrum of the main constructs deemed important and necessary for the development of clinical reasoning. However, inconsistencies in agreement about the underpinning conceptual framework for clinical reasoning were evident. Further, for 11 evaluation tools adopted for medical and nursing students, no framework was specified [59,60,61,62,63,64,65,66,67,68,69]. Many of these evaluation tools were described as rubrics, examinations, and objective structured clinical examinations (OSCEs), and the authors did not clearly define the construct being assessed as one of critical thinking, clinical judgement, clinical decision making, or clinical reasoning. It would appear that if a conceptual framework for clinical reasoning is used, then the specific constructs are identified and built into the assessment tool, whereas for those without a conceptual framework, it was found that the constructs of the evaluation tools are often only identified in general terms. These tools may, for example, purport to represent ‘clinical reasoning’ generically by way of a proxy activity such as clinical documentation.

## 4. Discussion

This review identified numerous tools used to evaluate clinical reasoning and related constructs in placements and simulation in health professional education. Of these tools, the Script Concordance Test [16] and Lasater Clinical Judgement Rubric [17] were prominent in the literature. The diversity of additional tools identified from searches using a range of terms provides educators with a variety of options for student evaluation in these situations. These tools encompass a spread of approaches along the ‘continuum of authenticity’ [5], given our inclusion of evaluation tools described as being conducted during, or associated with, clinical placement or simulation settings, even if not explicitly of workplace performance. However, there is a lack of cross-referencing between tools and constructs identified in this review, and evidence of continued development is almost exclusively within discipline boundaries. From identifying these tools and their conceptual foundations, we present four key implications for further discussion.

### 4.1. There Remains Inconsistent Use of Terminology around Clinical Reasoning

Unsurprisingly, given previous reports [2,3,4,6,7], there is a lack of consistency in the application of terminology to name the constructs being assessed. Different terminology appears preferred in different discipline groups, such as ‘critical thinking skills’ and ‘clinical judgement’ being a focus in nursing and midwifery, whereas ‘clinical reasoning’ is in more widespread use and appears often as a more general term [7]. The use of conceptual frameworks to explicate constructs is limited, and different frameworks are used to define the same constructs as in ‘critical thinking’ [18,19], with varied evaluation tools even from the same frameworks. Included among the tools to evaluate ‘clinical reasoning’ were evaluations of the application of specific frameworks for ‘reasoning’ that were not themselves models of clinical reasoning [32,34], diagnostic processes as in the Script Concordance Test, etc. [34,77], and therapeutic processes [57]. Finally, there was a group of papers that examined diverse evaluation tools of relevance to the topic but without clearly setting out the origin of the construct of interest, sometimes not even clearly naming a construct at all.

To advance both practice and research, there remains a need to clarify both terminology and constructs, and to achieve this in ways that will enable a better understanding of how each profession contributes similarly and differently to clinical reasoning in the practice of both diagnosis and management [3,7,94]. Communicating clearly about, and reconciling, conceptual and theoretical frameworks will be required to advance this cause [6], in the literature more broadly and in reference to the evaluation of clinical reasoning and related constructs specifically.

### 4.2. Each Evaluation Tool Has Limited Evidence

This review also highlighted that the evidence to support the use of evaluation tools or make choices between them is, in most cases, limited for these learning contexts. The Script Concordance Test [16] and Lasater Clinical Judgement Rubric [17] clearly dominated the published work, but there were many more tools reported on with very limited interconnections made between them or even cross-referencing of research.

It is striking that there was no overlap with measures of critical thinking identified in a previous systematic review of studies with experimental designs [9], and limited overlap with those identified in a systematic review of a broad range of measures used to assess simulation outcomes [10]. In part, the development of some of the tools in this review was in response to the need for appropriate outcome measures that prior reviews in specific circumstances have identified, but overwhelmingly, the evaluation tools in this review have been developed and tested in isolation of each other and with limited subsequent applications. The few papers per available tool offer limited evidence for evaluation of health professional students in clinical and simulated placements.

### 4.3. There Is Minimal Evidence for Allied Health or Multi-Disciplinary Crossover

Somewhat surprisingly, there were only 4 papers (of the total of 61) that considered tools for the evaluation of clinical reasoning in disciplines beyond nursing and medicine (i.e., allied health), which have been the focus of prior reviews [5,9]. The importance of clinical reasoning in allied health is similarly critical, with many allied health disciplines being primary care practitioners. Clinical reasoning is also important for patient management decisions in all these professions and for working in multidisciplinary teams. With the lack of research, it is unclear if findings on existing tools are applicable to allied health student development.

There also remains no identified avenues of considering how students from differing disciplines engage in clinical reasoning regarding the same patient scenario, with implications for teaching and evaluation of constructs [7], and also for understanding how healthcare teams may work together. A few studies have used the Script Concordance Test beyond medical specialties, with one study including pharmacy students and two including nursing students. However, it should be noted that the case vignettes for the Script Concordance Test were developed and calibrated against content expert decisions for each instance completely independently. Even single studies, where participants were drawn from multiple disciplines or medical specialties [89,91] used multiple instances of the test rather than a direct comparison. Again, this lack of multi-disciplinary crossover is reflective of the usage of terminology and reinforces the need to make explicit intended meanings [7]. If health professionals can speak the same language when considering clinical reasoning pedagogically and clinically, the impacts may be seen on student learning, collaborative decision making, and patient care.

### 4.4. Evaluation Tools Reported in the Literature Represent Two Contrasting Objectives

The two differing objectives of clinical reasoning measurement represented by the tools in this review, represent contrasts in emphasis on diagnostic versus management considerations in reasoning [94]. When tools are compared side by side, it is apparent that evaluating students’ clinical reasoning is complex. The tools identified do not, individually or even between them, represent the full complexity of the construct of reasoning set out in the literature [4,5,94]. Some approaches to student evaluation emphasise cognitive functioning, and perhaps specificity and objectivity, as do standardised psychometric measures of critical thinking abilities that have been used in non-clinical settings of health professional education [10]. Other approaches to student evaluation favour comprehensive coverage in practice situations. Each takes different approaches toward considering how students manage inherently dynamic healthcare situations. Given none is complete, educators must be mindful of how the application of different constructs when evaluating students’ learning can lead to different interpretations.

#### 4.4.1. Tools to Assess the Development of Diagnostic Reasoning

A key objective of assessment in a subset of the papers included in this review is to examine the congruence between students’ reasoning outcomes and those deemed as experts. This is evident predominantly in instances of the Script Concordance Test. This objective necessitates the identification of a point of clear comparison, which can restrict the aspects and applications of clinical reasoning that can be evaluated. In contrast to definitions of clinical reasoning, which incorporate the whole therapeutic process [2,4], the focus from this objective is typically on diagnosis and treatment decisions where agreement can be objectified. The expectation is that students start out using limited network approaches in knowledge organisation and build refined knowledge scripts with experience [95]. This can be a useful way to consider the development of expertise and evaluations using this approach, while usually being paper-based and closed-response evaluations, are able to track shifts in respondents’ thinking as new data informing clinical reasoning is provided. However, the domains covered need to be married with complementary approaches or comprehensive evaluation to encapsulate all components of clinical reasoning [5].

#### 4.4.2. Tools to Judge the Quality of Performance as a Reflection of Reasoning Processes

The alternate objective of assessment in the papers in this review is to judge the quality of performance of steps that apply throughout a process of therapeutic patient management. Such reasoning processes are clearly set out in models such as the Clinical Judgement Model [49] and Clinical Reasoning Process [51]. This gives rise to the labelling of this construct as ‘clinical judgement’ in tools such as the Lasater Clinical Judgement Rubric [17], while others [57,58] term a very similar construct as clinical reasoning. These instruments address multiple clinical reasoning components beyond diagnosis or scenario planning, which is more consistent with broad definitions of clinical reasoning [2,4,5]. They typically make use of the authentic and dynamic situations in simulation and clinical placements, relying on extended and preferably multiple observations of student reasoning performance with assessor judgement [5]. However, this approach may introduce the risk of misjudgement by assessors if using observable performance outputs (behaviours) to infer cognitive and affective elements of reasoning. It may also be difficult to distinguish between the cognitive, psychomotor, and affective skills in reasoning, and broader therapeutic or technical skills. For example, the Lasater Clinical Judgement Rubric includes ‘calm, confident manner’, ‘clear communication’, and ‘being skilful’ among the elements of clinical judgement [17]. Evaluations using this approach might, therefore, be more holistic than specific and sensitive to clinical reasoning and thereby student development.

## 5. Limitations

This systematic review sought to identify the range of tools reported in the literature for educators to consider in evaluating the clinical reasoning or related abilities of allied health and medical students in simulated or placement settings. This represents only the formally published, English-language literature on the topic, subject to publication bias and limited international representation, particularly as grey literature searching and secondary search strategies were not used (e.g., reference list and citation tracking, or contacting authors). Author reports were used rather than independent analysis of constructs and theoretical frameworks represented in evaluation tools, given the full content of many tools were not available, and thus, variation in the use of constructs and terms was visible but not resolved in this study. Nonetheless, this review was able to present a broad overview with respect to the inclusions of disciplines of the students while focussing on the evaluation of clinical reasoning and related constructs specifically.

## 6. Conclusions

This study identified a significant number of tools used to evaluate clinical reasoning and related constructs in placement and simulation settings in health professional education. There is a lack of cross-referencing between tools and constructs identified in this review, and evidence of continued development is only observed within discipline boundaries. Unfortunately, if disciplines do not share a common understanding of the conceptual framework or constructs of clinical reasoning, this may impact on and limit interprofessional learning and collaborative clinical judgements in multidisciplinary teams.

Future research into clinical reasoning evaluation tools should build on and reference existing approaches and consider contributions across professional disciplinary divides. Research is needed to develop, test, and incorporate student evaluations that are applicable to outcome measurement in research studies in order to understand students’ performance of this essential capability and how to support its development. A larger evidence base than was identified for most tools in this review is required for that purpose, with attention to research quality. Repeated measures and longitudinal perspectives capturing students’ reasoning development are specifically required, as are workplace-based approaches [14]. By connecting and expanding this body of work, it will be possible to more clearly identify contributors to students’ learning and their attainment of threshold skills. Clearly, more research is required to sequence the development of clinical reasoning by standardising the use of terminology and constructs and considering tool design that can monitor the developmental progression of clinical reasoning progression with applicability across health professions.

## Figures and Tables

**Table 1:** Inclusion and exclusion criteria.

Inclusion Criteria	Exclusion Criteria
Studies meeting ALL the following: Peer reviewed Published 2000-2018 Published in English Accessible to these authors Any health profession Pre-registration student education Clinical reasoning or related concepts Primary outcome is to develop or test an evaluation tool	Used but did not develop or test an evaluation tool Developed or tested an evaluation of a whole range of clinical skills or competencies, even if these included a component of clinical reasoning Evaluation tools only used outside of clinical placement or simulation settings

**Table 2:** Constructs measured in evaluations of students’ clinical reasoning.

Construct	Theoretical Underpinning	Evaluation Tool or Measure (Discipline of Student)
Clinical Decision Making	None stated (an adaptation of the Physical Therapist Clinical Performance Instrument) [28]	Clinical Decision Making Survey Tool (Physical Therapy) [29]
	Not stated	Surgical Decision Making Rating Scale (Medicine) [59]
Clinical Judgement	Clinical Judgement Model [49]	Lasater Clinical Judgement Rubric (Nursing) [17,70,71,72,73,74,75,76]
		Lasater Clinical Judgement Rubric—Korean version (Nursing) [52]
		Lasater Clinical Judgement Rubric—Dutch version (Nursing) [53]
		Virtual Patient Lasater Clinical Judgement Rubric (Nursing) [54]
		Scenario-specific Assessment Tool for Febrile Infant Care Simulation (adaptation of the Lasater Clinical Judgement Rubric; Nursing) [55]
		Simulation Evaluation Tool (an adaptation of the Lasater Clinical Judgement Rubric) (Nursing) [56]
Clinical Reasoning	“A SECRET” reasoning approach [33]	A SECRET Assessment (Occupational Therapy) [32]
	Clinical Reasoning Process model [51]	Clinical Reasoning Evaluation Simulation Tool (CREST) (Nursing) [57]
		Nurses Clinical Reasoning Scale (Nursing) [58]
	IDEA Framework, structural semantics, and RIME [35]	IDEAs Assessment Tool (Medicine) [34]
	Novice Clinical Reasoning Model [45] and Social cognitive theory [46,47]	Nursing Anxiety and Self-Confidence with Clinical Decision Making (NASC-CDM; Nursing) [48]
	Outcome Present State Test Model [50]	Outcome Present State Test (OPT; Nursing) [77]
	Revised Bloom’s Taxonomy [44] and Dreyfus Model [13]	Clinical Reasoning Grading Rubric (Physical Therapy) [43]
	Script Theories [11,12]	Multiple Choice Question Exam (Medicine) [36] *
		Script Concordance Test (Medicine) [36] *, [78,79,80,81,82,83,84,85,86,87,88,89], [90] *, [91] * (Nursing) [92], [91] * (Pharmacy) [93]
		Script Concordance Test with Think Aloud (Medicine) [37], [90] *
	Not stated	Clinical Reasoning Problems Test (Medicine) [60], [90] *
Critical Thinking	Benner’s [41] levels of nursing experience, and Bloom’s [42] cognitive domains	Clark Simulation Evaluation Rubric (Nursing) [40]
	Consensus dimensions of critical thinking in nursing [18]	Carter Assessment of Critical Thinking in Midwifery (Preceptor / Mentor Version) (Midwifery) [21], [23] *
		Carter Assessment of Critical Thinking in Midwifery (Student Self-Rating Version) (Midwifery) [22], [23] *
		Carter Assessment of Critical Thinking in Midwifery (Reflective Writing) (Midwifery) [23] *
		Rubric for assessing critical thinking dimensions (Nursing) [20]
	Expert Consensus on Critical Thinking [19]	Critical Thinking Self-Reflection Tool (Nursing) [24]
		Yoon’s Critical Thinking Tool (Nursing) [25]
	IDEAS five-step critical thinking problem-solving process [31]	Critical Thinking Skills Rating Instrument (CTSRI; Medicine) [30]
	Structured Observation of and Assessment of Practice [27]	Clinical Viva (Nursing) [26]
Situation Awareness	Situation Awareness [38]	Situation Awareness Global Assessment Technique (SAGAT; Nursing) [39]
Not Specified	Not stated	Clinical Performance Examination (CPX; Medicine) [61]
		Computer-based Case Simulation (CCS; DxR Clinician Software; Medicine) [63]
		Exam formats: Context-rich single best answer versus key feature problems (Medicine) [62]
		Exam formats: Extended matching questions, with think aloud (Medicine) [64]
		Interactive Simulation of Patients Objective Structured Clinical Examination (OSCE) Station (Medicine) [65]
		Objective Structured Clinical Examination (OSCE) Note Writing Station (Medicine) [66]
		Reflective Thinking Instrument (Nursing) [67]
		Virtual Patient Case Patient Summary Statement Rubric (Medicine) [68]
		Virtual Patient Case Procedural Rubric and Semantic Rubric (Medicine) [69]

Note: Papers marked with * included more than one assessment and are thereby included more than once.

## Data Availability

The included papers are referenced within, and data extraction tables are included within and in Supplementary Materials. A full list of identified references including all those excluded from the study is available from the corresponding author on request.

## Supplementary Materials

The following are available online at https://www.mdpi.com/article/10.3390/ijerph19020936/s1, Table S1: Search strategy, Table S2: Tools for evaluating students’ clinical reasoning.
