# Supplementary material for: Evaluating the Clinical Reasoning of Student Health Professionals in Placement and Simulation Settings: A Systematic Review
Source: Int J Environ Res Public Health. 2022 Jan 14;19(2):936. doi: 10.3390/ijerph19020936 (PMC8775520; doi:10.3390/ijerph19020936)
Supplement: Supplementary file 1 [file ijerph-19-00936-s001.zip › ijerph-1509877-supplementary.pdf]

International Journal of
Environmental Research and Public Health

MDPI

Supplementary Table S1. Search strategy.

	**Health profession**	**AND Student**	**AND Reasoning**	**AND Tool Development and Testing**
CINAHL (via EBSCO)	"allied health profession*" or "allied health therap*" or "physiotherap*" or "physical therap*" or "occupational therap*" or "social work*" or "speech patholog*" or "speech therap*" or "speech language*" or "dietician*" or "dietetic*" or "podiatr*" or "audiolog*" or "psychol*" or "pharma*" or "exercise physiolog*" or "paramedic*" or "nurs*" or "medical student*"	"student"	"reasoning" or "critical thinking" or "judgement" or "problem solving" or "decision making"	"inventory" or "assessment" or "evaluation" or "rubric" or "test" or "scale" or "measure" or "index" or "examination" or "survey" or "questionnaire" or (MH "Educational Measurement+")
EMBASE, ERIC, Medline, **Premedline** and PsychINFO (via Ovid)	allied health profession*.mp or allied health therap*.mp or physiotherap*.mp or physical therap*.mp or occupational therap*.mp or social work*.mp or speech patholog*.mp or speech therap*.mp or speech language patholog*.mp or speech language therap*.mp or dietician.mp or dietetic*.mp or podiatr*.mp or audiolog*.mp or psychol*.mp or pharma*.mp or exercise physiolog*.mp or paramedic*.mp or nurs*.mp or medical student*.mp	Student.mp	reasoning.mp or critical thinking.mp or judgement.mp or problem solving/ or decision making/	Inventory.mp or Assessment.mp or Evaluation.mp or Rubric.mp or Test.mp or Scale.mp or Measure.mp or Index.mp or Examination.mp or "Surveys and questionnaires"/ or Educational measurement/
Proquest Nursing and Allied Health	noft("allied health profession*" or "allied health therap*" or "physiotherap*" or "physical therap*" or "occupational therap*" or "social work*" or "speech patholog*" or "speech therap*" or "speech language*" or "dietician*" or "dietetic*" or "podiatr*" or "audiolog*" or "psychol*" or "pharma*" or "exercise physiolog*" or "paramedic*" or "nurs*" or "medical student*")	noft(student)	noft("reasoning" or "critical thinking" or "judgement" or "problem solving" or "decision making")	noft("inventory" or "assessment" or "evaluation" or "rubric" or "test" or "scale" or "measure" or "index" or "examination" or "survey" or "questionnaire")

Supplementary Table S2. Tools for evaluating students' clinical reasoning.

Evaluation Tool or Measure	Paper First Author[1], Year	Country of Study	Discipline of Learner
A SECRET Assessment	Gee, 2017 [32]	USA	Occupational Therapy
Clark Simulation Evaluation Rubric	Gantt, 2010 [40]	USA	Nursing
Clinical Reasoning Evaluation Simulation Tool (CREST)	Liaw, 2018 [57]	Australia (+ international)	Nursing
Carter Assessment of Critical Thinking in Midwifery (Preceptor / Mentor Version)	Carter, 2016 [21]<br>Carter, 2018 [23]*	Australia	Midwifery
Carter Assessment of Critical Thinking in Midwifery (Student Self-Rating Version)	Carter, 2017 [22]<br>Carter, 2018 [23]*	Australia	Midwifery
Carter Assessment of Critical Thinking in Midwifery (Reflective Writing)	Carter, 2018 [23]*	Australia	Midwifery
Clinical Decision Making Survey Tool	Brudvig, 2017 [29]	USA	Physical therapy
Clinical Performance Examination (CPX)	Im, 2016 [61]	Korea	Medicine (n.s.)
Clinical Reasoning Problems Test	Derakhshandeh, 2018 [60]	Iran	Medicine (Cardiology)
	Groves, 2013 [90]*	Australia	Medicine (GP)
Computer-based Case Simulation (CCS; DxR Clinician Software)	Fida, 2015 [63]	Bahrain	Medicine (n.s.)
Critical Thinking Self-Reflection Tool	Cise, 2004 [24]	USA	Nursing
Critical Thinking Skills Rating Instrument (CTSRI)	Nguyen, 2017 [30]	USA	Medicine (n.s.)
Exam format: Context-rich single best answer versus key feature problems	Huwendiek, 2017 [62]	Germany	Medicine (Paediatrics)
Exam format: Extended matching questions (with think aloud)	Beullens, 2005 [64]	Belgium	Medicine (Various)
Exam format: Multiple Choice Question	Kelly, 2012 [36]*	USA	Medicine (Internal)
IDEAs Assessment Tool	Baker, 2015 [34]	USA	Medicine (n.s.)
Lasater Clinical Judgement Rubric	Adamson, 2012 [71]<br>Adamson, 2016 [70]<br>Ashcraft, 2013 [72]<br>Bussard, 2018 [73]<br>Lasater, 2007 [17]<br>Manetti, 2018 [74]<br>Strickland, 2017 [75]	USA	Nursing

Evaluation Tool or Measure	Paper First Author[1], Year	Country of Study	Discipline of Learner
	Román-Cereto, 2018 [76]	Spain	Nursing
Lasater Clinical Judgement Rubric – Dutch version	Vreugdenhil, 2018 [53]	The Netherlands	Nursing
Lasater Clinical Judgement Rubric – Korean version	Shin, Park & Shim, 2015 [52]	Korea	Nursing
Lasater Clinical Judgement Rubric – Virtual Patient	Georg, 2018 [54]	Sweden	Nursing
Lasater Clinical Judgement Rubric adaptation: Scenario-specific Assessment Tool for Febrile Infant Care Simulation	Shin, Shim, Lee & Quinn, 2014 [55]	Korea	Nursing
Lasater Clinical Judgement Rubric adaptation: Simulation Evaluation Tool	Kim, 2016 [56]	Korea	Nursing
Nurses Clinical Reasoning Scale	Liou, 2016 [58]	Taiwan	Nursing
Nursing Anxiety and Self-Confidence with Clinical Decision Making (NASC-CDM)	White, 2014 [48]	USA	Nursing
Objective Structured Clinical Examination (OSCE) Station with Interactive Simulation of Patients	Courteille, 2008 [65]	Sweden	Medicine (Surgery)
Objective Structured Clinical Examination (OSCE) Station – Note Writing	Berger, 2012 [66]	USA	Medicine (n.s.)
Outcome Present State Test (OPT)	Kautz, 2009 [77]	USA	Nursing
Reflective Thinking Instrument	Tutticci, 2016 [67]	Australia	Nursing
Rubric, Clinical Reasoning Grading	Furze, 2015 [43]	USA	Physical therapy
Rubric, Critical Thinking Dimensions	Allen, 2004 [20]	USA	Nursing
Rubric, Virtual Patient Case Patient Summary Statement	Smith, 2016 [68]	USA	Medicine (Various)
Rubric, Virtual Patient Case Procedural Rubric and Semantic	Fleiszer, 2018 [69]	Canada	Medicine (Surgery)
Surgical Decision Making Rating Scale	Chatterjee, 2009 [59]	Canada	Medicine (Urology)
Script Concordance Test	Amini, 2017 [78]	Iran	Medicine (Internal)
	Boulouffe, 2014 [79]	Canada	Medicine (Emergency)
	Dawson, 2014 [92]	USA	Nursing
	Funk, 2017 [93]	USA	Pharmacy
	Gagnon, 2009 [91]	Canada	Medicine (Radiation Oncology & Paediatrics) & Nursing
	Goos, 2016 [80]	Germany	Medicine (Surgery)
	Groves, 2013 [90]*	Australia	Medicine (GP)

**Evaluation Tool or Measure**	**Paper First Author[1], Year**	**Country of Study**	**Discipline of Learner**
	Humbert, 2011 [81]	USA	Medicine (Emergency)
	Kania, 2011 [82]	France	Medicine (ENT)
	Kazour, 2017 [83]	France	Medicine (Psychiatry)
	Kelly, 2012 [36]*	USA	Medicine (Internal)
	Lambert, 2009 [84]	Canada	Medicine (Radiation Oncology)
	Lubarsky, 2009 [96]	Canada	Medicine (Neurology)
	Ruiz, 2010 [85]	USA	Medicine (Internal/ Geriatrics)
	Sibert, Charlin et al., 2002 [86]<br>Sibert, Darmoni et al., 2006 [87]	France	Medicine (Urology)
	Subra, 2017 [88]	France	Medicine (GP)
	Wan, 2018 [89]	Australia	Medicine (Various)
Script Concordance Test with Think Aloud	Power, 2017 [37]	Canada	Medicine (Paediatrics)
	Groves, 2013 [90]*	Australia	Medicine (GP)
Situation Awareness Global Assessment Technique (SAGAT)	Lavoie, 2016 [39]	Canada	Nursing
Viva, clinical	Roberts, 2013 [26]	UK	Nursing
Yoon's Critical Thinking Tool	Shin, Park & Kim, 2015 [25]	Korea	Nursing
